# 2-(4-Methyl­phen­yl)-5-[({[5-(4-methyl­phen­yl)-1,3,4-thia­diazol-2-yl]sulfan­yl}meth­yl)sulfan­yl]-1,3,4-thia­diazole

**DOI:** 10.1107/S1600536812007520

**Published:** 2012-02-24

**Authors:** Yong Wang, Wen-ge Zhang, Yu-bo Wang, Jing-wen Yu, Lin Zhou

**Affiliations:** a School of Chemical Engineering, University of Science and Technology LiaoNing, Anshan 114051, People’s Republic of China; b Anshan Normal University, Anshan 114005, People’s Republic of China; c Shengyang Agricultural University, Shengyang 116121, People’s Republic of China

## Abstract

In the title compound, C_19_H_16_N_4_S_4_, the mol­ecules exhibit a butterfly conformation, where the thia­diazole and attached benzene rings in two wings are almost coplanar, with dihedral angles of 0.8 (3) and 0.9 (3)°, respectively, while the two thia­diazole rings form a dihedral angle of 46.3 (3)°.

## Related literature

For the biological properties of 1,3,4-thia­diazole derivatives, see: Nakagawa *et al.* (1996 ▶); Wang *et al.* (1999 ▶); Carvalho *et al.* (2004 ▶). For the crystal structures of related compounds, see: Li *et al.* (2011 ▶); Wang *et al.* (2010 ▶).
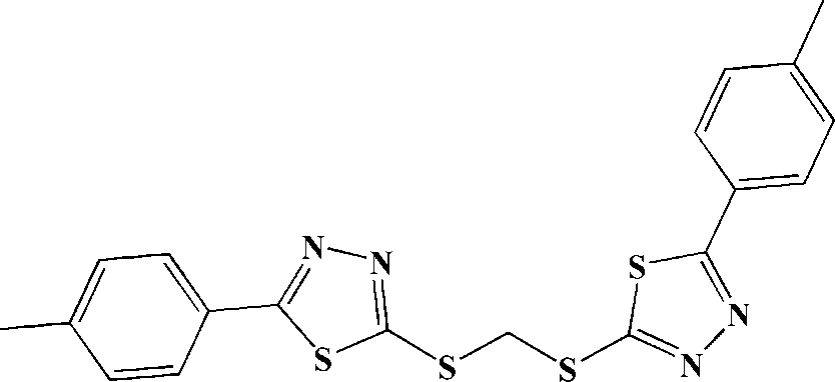


## Experimental

### 

#### Crystal data

C_19_H_16_N_4_S_4_
*M* *_r_* = 428.60
Monoclinic, 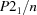

*a* = 16.8944 (14) Å
*b* = 4.1959 (5) Å
*c* = 27.107 (2) Å
β = 96.084 (8)°
*V* = 1910.7 (3) Å^3^
*Z* = 4
Mo *K*α radiation
μ = 0.51 mm^−1^
*T* = 113 K
0.50 × 0.04 × 0.04 mm

#### Data collection

Rigaku Saturn CCD area-detector diffractometer
Absorption correction: multi-scan (*CrystalClear*; Rigaku/MSC, 2005 ▶) *T*_min_ = 0.785, *T*_max_ = 0.980
14969 measured reflections
4535 independent reflections
2927 reflections with *I* > 2σ(*I*)
*R*_int_ = 0.094

#### Refinement

*R*[*F* ^2^ > 2σ(*F* ^2^)] = 0.057
*wR*(*F* ^2^) = 0.142
*S* = 0.96
4535 reflections
246 parameters
H-atom parameters constrained
Δρ_max_ = 0.42 e Å^−3^
Δρ_min_ = −0.60 e Å^−3^

### 

Data collection: *CrystalClear* (Rigaku/MSC, 2005 ▶); cell refinement: *CrystalClear*; data reduction: *CrystalClear*; program(s) used to solve structure: *SHELXS97* (Sheldrick, 2008 ▶); program(s) used to refine structure: *SHELXL97* (Sheldrick, 2008 ▶); molecular graphics: *SHELXTL* (Sheldrick, 2008 ▶); software used to prepare material for publication: *SHELXL97*.

## Supplementary Material

Crystal structure: contains datablock(s) global, I. DOI: 10.1107/S1600536812007520/cv5245sup1.cif

Structure factors: contains datablock(s) I. DOI: 10.1107/S1600536812007520/cv5245Isup2.hkl

Supplementary material file. DOI: 10.1107/S1600536812007520/cv5245Isup3.cml

Additional supplementary materials:  crystallographic information; 3D view; checkCIF report

## Figures and Tables

**Fig. 1.**
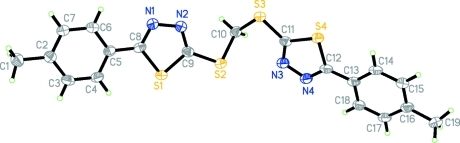
View of (I) showing the atom-labelling scheme. Displacement ellipsoids are drawn at the 60% probability level.

## supplementary crystallographic information

### Comment

1,3,4-Thiadiazole derivatives are important for medicinal chemistry due to their chemical and pharmaceutical properties (Nakagawa *et al.*, 1996; Wang *et al.*, 1999; Carvalho *et al.*, 2004). Similar crystal structures of the 1,3,4-thiadiazole derivatives have been reported . As a part of our research, the title compound (I) has been synthesized, and herewith we present its crystal structure.

In (I) (Fig. 1), all geometric parameters are normal and comparable with those reported for related 1,3,4-thiadiazole derivatives (Li *et al.*, 2011; Wang *et al.*, 2010). Two thiadiazole rings form a dihedral angle of 46.3 (3)%. The dihedral angles between the benzene rings and attached thiadiazole rings are 0.8 (3) and 0.9 (3)° indicating the two rings are almost parallel. The same situation has been observed in the crystal structure of 1,4-bis(5-phenyl-1,3,4-thiadiazol-2-ylsulfanyl)butane (Li *et al.*, 2011).

### Experimental

The title compound was synthesized by the reaction of the 1,1-dibromomethane (1.0 mmol) and 5-tolyl-1,3,4-thiadiazol-2-thiol (2.0 mmol) in ethanol (20 ml) at room temperature. Crystals of (I) suitable for single-crystal X-ray analysis were grown by slow evaporation of a solution in chloroform-enthanol (1:1).

### Refinement

All H atoms were positioned geometrically and refined as riding (C—H = 0.95 - 0.99 Å), with *U*_iso_(H) = 1.2 - 1.5*U*_eq_ (C).

### Crystal data

**Table:**

C_19_H_16_N_4_S_4_	*F*(000) = 888
*M**_r_* = 428.60	*D*_x_ = 1.490 Mg m^−^^3^
Monoclinic, *P*2_1_/*n*	Mo *K*α radiation, λ = 0.71073 Å
Hall symbol: -P 2yn	Cell parameters from 5461 reflections
*a* = 16.8944 (14) Å	θ = 1.4–27.8°
*b* = 4.1959 (5) Å	µ = 0.51 mm^−^^1^
*c* = 27.107 (2) Å	*T* = 113 K
β = 96.084 (8)°	Prism, colourless
*V* = 1910.7 (3) Å^3^	0.50 × 0.04 × 0.04 mm
*Z* = 4	

### Data collection

**Table:**

Rigaku Saturn CCD area-detector diffractometer	4535 independent reflections
Radiation source: rotating anode	2927 reflections with *I* > 2σ(*I*)
Multilayer monochromator	*R*_int_ = 0.094
Detector resolution: 14.22 pixels mm^-1^	θ_max_ = 27.8°, θ_min_ = 1.4°
φ and ω scans	*h* = −22→22
Absorption correction: multi-scan (*CrystalClear*; Rigaku/MSC, 2005)	*k* = −5→4
*T*_min_ = 0.785, *T*_max_ = 0.980	*l* = −35→31
14969 measured reflections	

### Refinement

**Table:**

Refinement on *F*^2^	Primary atom site location: structure-invariant direct methods
Least-squares matrix: full	Secondary atom site location: difference Fourier map
*R*[*F*^2^ > 2σ(*F*^2^)] = 0.057	Hydrogen site location: inferred from neighbouring sites
*wR*(*F*^2^) = 0.142	H-atom parameters constrained
*S* = 0.96	*w* = 1/[σ^2^(*F*_o_^2^) + (0.0566*P*)^2^] where *P* = (*F*_o_^2^ + 2*F*_c_^2^)/3
4535 reflections	(Δ/σ)_max_ = 0.001
246 parameters	Δρ_max_ = 0.42 e Å^−^^3^
0 restraints	Δρ_min_ = −0.60 e Å^−^^3^

### Special details

**Table:**

Geometry. All e.s.d.'s (except the e.s.d. in the dihedral angle between two l.s. planes) are estimated using the full covariance matrix. The cell e.s.d.'s are taken into account individually in the estimation of e.s.d.'s in distances, angles and torsion angles; correlations between e.s.d.'s in cell parameters are only used when they are defined by crystal symmetry. An approximate (isotropic) treatment of cell e.s.d.'s is used for estimating e.s.d.'s involving l.s. planes.
Refinement. Refinement of *F*^2^ against ALL reflections. The weighted *R*-factor *wR* and goodness of fit *S* are based on *F*^2^, conventional *R*-factors *R* are based on *F*, with *F* set to zero for negative *F*^2^. The threshold expression of *F*^2^ > σ(*F*^2^) is used only for calculating *R*-factors(gt) *etc*. and is not relevant to the choice of reflections for refinement. *R*-factors based on *F*^2^ are statistically about twice as large as those based on *F*, and *R*- factors based on ALL data will be even larger.

### Fractional atomic coordinates and isotropic or equivalent isotropic displacement parameters (Å^2^)

**Table:**

	*x*	*y*	*z*	*U*_iso_*/*U*_eq_	
S1	0.41711 (5)	0.77165 (19)	−0.01333 (3)	0.0266 (2)	
S2	0.39123 (5)	1.1072 (2)	0.08165 (3)	0.0282 (2)	
S3	0.26282 (5)	1.06832 (19)	0.15267 (3)	0.0270 (2)	
S4	0.33462 (5)	1.07934 (19)	0.26002 (3)	0.0268 (2)	
N1	0.26848 (16)	0.6479 (6)	−0.02748 (10)	0.0285 (6)	
N2	0.27897 (16)	0.8187 (6)	0.01681 (10)	0.0288 (6)	
N3	0.39960 (17)	1.3525 (6)	0.18983 (10)	0.0292 (6)	
N4	0.44931 (16)	1.4137 (6)	0.23312 (10)	0.0289 (6)	
C1	0.3546 (2)	−0.0838 (8)	−0.23078 (12)	0.0349 (8)	
H1A	0.3223	−0.2782	−0.2313	0.052*	
H1B	0.4101	−0.1403	−0.2341	0.052*	
H1C	0.3343	0.0547	−0.2584	0.052*	
C2	0.3500 (2)	0.0891 (7)	−0.18231 (12)	0.0263 (7)	
C3	0.4179 (2)	0.2240 (8)	−0.15616 (12)	0.0296 (8)	
H3	0.4680	0.2007	−0.1687	0.036*	
C4	0.4135 (2)	0.3902 (7)	−0.11251 (12)	0.0283 (7)	
H4	0.4602	0.4807	−0.0955	0.034*	
C5	0.34068 (19)	0.4251 (7)	−0.09335 (11)	0.0237 (7)	
C6	0.27246 (19)	0.2897 (7)	−0.11872 (12)	0.0268 (7)	
H6	0.2225	0.3126	−0.1060	0.032*	
C7	0.2776 (2)	0.1227 (7)	−0.16224 (12)	0.0275 (7)	
H7	0.2309	0.0289	−0.1788	0.033*	
C8	0.33463 (19)	0.6019 (7)	−0.04729 (11)	0.0242 (7)	
C9	0.35275 (19)	0.8994 (7)	0.02832 (11)	0.0255 (7)	
C10	0.2996 (2)	1.2661 (7)	0.10072 (12)	0.0277 (7)	
H10A	0.3080	1.4939	0.1092	0.033*	
H10B	0.2581	1.2551	0.0721	0.033*	
C11	0.33781 (19)	1.1814 (7)	0.19861 (12)	0.0247 (7)	
C12	0.42392 (18)	1.2861 (7)	0.27270 (12)	0.0258 (7)	
C13	0.46511 (19)	1.3186 (7)	0.32295 (12)	0.0262 (7)	
C14	0.43531 (19)	1.1764 (8)	0.36363 (12)	0.0278 (7)	
H14	0.3872	1.0580	0.3591	0.033*	
C15	0.4755 (2)	1.2068 (8)	0.41067 (12)	0.0292 (7)	
H15	0.4540	1.1096	0.4380	0.035*	
C16	0.54653 (19)	1.3758 (7)	0.41906 (12)	0.0285 (7)	
C17	0.5753 (2)	1.5225 (8)	0.37799 (12)	0.0286 (7)	
H17	0.6231	1.6434	0.3826	0.034*	
C18	0.53548 (19)	1.4947 (8)	0.33077 (12)	0.0279 (7)	
H18	0.5562	1.5961	0.3035	0.033*	
C19	0.5928 (2)	1.3981 (9)	0.46959 (12)	0.0346 (8)	
H19A	0.6398	1.2602	0.4708	0.052*	
H19B	0.5590	1.3294	0.4949	0.052*	
H19C	0.6096	1.6190	0.4760	0.052*	

### Atomic displacement parameters (Å^2^)

**Table:**

	*U*^11^	*U*^22^	*U*^33^	*U*^12^	*U*^13^	*U*^23^
S1	0.0174 (4)	0.0334 (5)	0.0289 (4)	−0.0015 (3)	0.0025 (3)	−0.0022 (4)
S2	0.0207 (4)	0.0359 (5)	0.0279 (4)	−0.0021 (3)	0.0019 (3)	−0.0029 (4)
S3	0.0204 (4)	0.0320 (5)	0.0285 (4)	−0.0002 (3)	0.0022 (3)	−0.0018 (3)
S4	0.0197 (4)	0.0329 (5)	0.0279 (4)	−0.0018 (3)	0.0022 (3)	0.0007 (3)
N1	0.0230 (15)	0.0343 (16)	0.0279 (14)	0.0008 (12)	0.0014 (12)	−0.0048 (12)
N2	0.0242 (15)	0.0342 (16)	0.0282 (14)	0.0000 (12)	0.0035 (12)	−0.0044 (12)
N3	0.0228 (15)	0.0352 (16)	0.0296 (14)	−0.0016 (12)	0.0026 (12)	0.0011 (12)
N4	0.0211 (15)	0.0357 (16)	0.0297 (15)	−0.0025 (12)	0.0013 (12)	0.0020 (12)
C1	0.034 (2)	0.037 (2)	0.0336 (19)	0.0041 (16)	0.0044 (16)	−0.0033 (16)
C2	0.0266 (18)	0.0262 (17)	0.0261 (16)	0.0032 (14)	0.0024 (14)	0.0034 (13)
C3	0.0204 (17)	0.0360 (19)	0.0332 (18)	0.0014 (14)	0.0064 (15)	0.0030 (15)
C4	0.0199 (17)	0.0312 (18)	0.0332 (18)	−0.0029 (14)	0.0003 (14)	−0.0010 (14)
C5	0.0209 (17)	0.0231 (16)	0.0262 (16)	0.0013 (13)	−0.0009 (13)	0.0034 (13)
C6	0.0203 (17)	0.0292 (17)	0.0311 (17)	0.0002 (13)	0.0035 (14)	0.0023 (14)
C7	0.0214 (17)	0.0306 (18)	0.0303 (17)	−0.0017 (14)	0.0012 (14)	−0.0005 (14)
C8	0.0196 (16)	0.0231 (16)	0.0290 (16)	0.0002 (13)	−0.0020 (13)	0.0030 (13)
C9	0.0230 (17)	0.0279 (17)	0.0261 (16)	0.0001 (14)	0.0045 (14)	0.0043 (14)
C10	0.0249 (18)	0.0283 (17)	0.0290 (17)	0.0051 (14)	−0.0019 (14)	−0.0024 (14)
C11	0.0185 (16)	0.0265 (17)	0.0291 (16)	0.0049 (13)	0.0034 (13)	0.0005 (13)
C12	0.0151 (16)	0.0272 (17)	0.0355 (18)	0.0012 (13)	0.0041 (14)	−0.0025 (14)
C13	0.0169 (16)	0.0276 (17)	0.0342 (18)	0.0044 (13)	0.0029 (14)	−0.0012 (14)
C14	0.0156 (16)	0.0309 (18)	0.0371 (18)	0.0004 (13)	0.0028 (14)	−0.0020 (15)
C15	0.0217 (17)	0.0355 (19)	0.0308 (17)	−0.0002 (14)	0.0044 (14)	−0.0010 (15)
C16	0.0193 (17)	0.0306 (18)	0.0350 (18)	0.0046 (14)	0.0001 (14)	−0.0035 (15)
C17	0.0169 (16)	0.0317 (18)	0.0373 (19)	0.0002 (14)	0.0027 (14)	−0.0037 (15)
C18	0.0210 (17)	0.0300 (17)	0.0329 (17)	0.0012 (14)	0.0043 (14)	−0.0028 (15)
C19	0.0221 (18)	0.046 (2)	0.0344 (18)	0.0029 (15)	−0.0023 (15)	−0.0011 (16)

### Geometric parameters (Å, º)

**Table:**

S1—C9	1.733 (3)	C4—H4	0.9500
S1—C8	1.739 (3)	C5—C6	1.398 (4)
S2—C9	1.753 (3)	C5—C8	1.465 (4)
S2—C10	1.810 (3)	C6—C7	1.383 (4)
S3—C11	1.745 (3)	C6—H6	0.9500
S3—C10	1.801 (3)	C7—H7	0.9500
S4—C11	1.725 (3)	C10—H10A	0.9900
S4—C12	1.743 (3)	C10—H10B	0.9900
N1—C8	1.304 (4)	C12—C13	1.469 (4)
N1—N2	1.393 (4)	C13—C14	1.394 (4)
N2—C9	1.297 (4)	C13—C18	1.397 (4)
N3—C11	1.310 (4)	C14—C15	1.386 (4)
N3—N4	1.393 (4)	C14—H14	0.9500
N4—C12	1.311 (4)	C15—C16	1.391 (4)
C1—C2	1.510 (4)	C15—H15	0.9500
C1—H1A	0.9800	C16—C17	1.403 (5)
C1—H1B	0.9800	C16—C19	1.506 (4)
C1—H1C	0.9800	C17—C18	1.386 (5)
C2—C7	1.398 (4)	C17—H17	0.9500
C2—C3	1.403 (4)	C18—H18	0.9500
C3—C4	1.382 (4)	C19—H19A	0.9800
C3—H3	0.9500	C19—H19B	0.9800
C4—C5	1.393 (4)	C19—H19C	0.9800
			
C9—S1—C8	87.06 (15)	S1—C9—S2	119.18 (19)
C9—S2—C10	99.51 (15)	S3—C10—S2	115.46 (17)
C11—S3—C10	98.52 (15)	S3—C10—H10A	108.4
C11—S4—C12	87.24 (15)	S2—C10—H10A	108.4
C8—N1—N2	113.2 (3)	S3—C10—H10B	108.4
C9—N2—N1	111.9 (3)	S2—C10—H10B	108.4
C11—N3—N4	111.6 (3)	H10A—C10—H10B	107.5
C12—N4—N3	113.2 (3)	N3—C11—S4	114.7 (2)
C2—C1—H1A	109.5	N3—C11—S3	123.5 (2)
C2—C1—H1B	109.5	S4—C11—S3	121.77 (18)
H1A—C1—H1B	109.5	N4—C12—C13	123.9 (3)
C2—C1—H1C	109.5	N4—C12—S4	113.2 (2)
H1A—C1—H1C	109.5	C13—C12—S4	123.0 (2)
H1B—C1—H1C	109.5	C14—C13—C18	118.6 (3)
C7—C2—C3	117.7 (3)	C14—C13—C12	121.2 (3)
C7—C2—C1	121.0 (3)	C18—C13—C12	120.1 (3)
C3—C2—C1	121.3 (3)	C15—C14—C13	120.3 (3)
C4—C3—C2	121.4 (3)	C15—C14—H14	119.8
C4—C3—H3	119.3	C13—C14—H14	119.8
C2—C3—H3	119.3	C14—C15—C16	121.9 (3)
C3—C4—C5	120.2 (3)	C14—C15—H15	119.1
C3—C4—H4	119.9	C16—C15—H15	119.1
C5—C4—H4	119.9	C15—C16—C17	117.3 (3)
C4—C5—C6	119.2 (3)	C15—C16—C19	122.4 (3)
C4—C5—C8	121.0 (3)	C17—C16—C19	120.3 (3)
C6—C5—C8	119.8 (3)	C18—C17—C16	121.4 (3)
C7—C6—C5	120.2 (3)	C18—C17—H17	119.3
C7—C6—H6	119.9	C16—C17—H17	119.3
C5—C6—H6	119.9	C17—C18—C13	120.4 (3)
C6—C7—C2	121.3 (3)	C17—C18—H18	119.8
C6—C7—H7	119.3	C13—C18—H18	119.8
C2—C7—H7	119.3	C16—C19—H19A	109.5
N1—C8—C5	124.5 (3)	C16—C19—H19B	109.5
N1—C8—S1	113.2 (2)	H19A—C19—H19B	109.5
C5—C8—S1	122.3 (2)	C16—C19—H19C	109.5
N2—C9—S1	114.6 (2)	H19A—C19—H19C	109.5
N2—C9—S2	126.2 (3)	H19B—C19—H19C	109.5
			
C8—N1—N2—C9	1.0 (4)	C11—S3—C10—S2	67.8 (2)
C11—N3—N4—C12	0.4 (4)	C9—S2—C10—S3	104.05 (19)
C7—C2—C3—C4	−1.3 (5)	N4—N3—C11—S4	−0.1 (3)
C1—C2—C3—C4	178.2 (3)	N4—N3—C11—S3	178.8 (2)
C2—C3—C4—C5	0.4 (5)	C12—S4—C11—N3	−0.2 (3)
C3—C4—C5—C6	0.1 (5)	C12—S4—C11—S3	−179.1 (2)
C3—C4—C5—C8	−179.6 (3)	C10—S3—C11—N3	−2.0 (3)
C4—C5—C6—C7	0.2 (5)	C10—S3—C11—S4	176.80 (19)
C8—C5—C6—C7	179.9 (3)	N3—N4—C12—C13	−179.3 (3)
C5—C6—C7—C2	−1.0 (5)	N3—N4—C12—S4	−0.6 (3)
C3—C2—C7—C6	1.6 (5)	C11—S4—C12—N4	0.4 (2)
C1—C2—C7—C6	−177.9 (3)	C11—S4—C12—C13	179.2 (3)
N2—N1—C8—C5	178.9 (3)	N4—C12—C13—C14	−179.8 (3)
N2—N1—C8—S1	−0.9 (3)	S4—C12—C13—C14	1.6 (4)
C4—C5—C8—N1	179.1 (3)	N4—C12—C13—C18	0.3 (5)
C6—C5—C8—N1	−0.7 (5)	S4—C12—C13—C18	−178.3 (2)
C4—C5—C8—S1	−1.2 (4)	C18—C13—C14—C15	−0.8 (5)
C6—C5—C8—S1	179.1 (2)	C12—C13—C14—C15	179.3 (3)
C9—S1—C8—N1	0.4 (2)	C13—C14—C15—C16	−0.5 (5)
C9—S1—C8—C5	−179.4 (3)	C14—C15—C16—C17	1.5 (5)
N1—N2—C9—S1	−0.7 (3)	C14—C15—C16—C19	−177.1 (3)
N1—N2—C9—S2	−178.4 (2)	C15—C16—C17—C18	−1.3 (5)
C8—S1—C9—N2	0.2 (3)	C19—C16—C17—C18	177.4 (3)
C8—S1—C9—S2	178.1 (2)	C16—C17—C18—C13	0.0 (5)
C10—S2—C9—N2	−16.9 (3)	C14—C13—C18—C17	1.0 (5)
C10—S2—C9—S1	165.53 (19)	C12—C13—C18—C17	−179.1 (3)
